# Status of gastrointestinal parasites in Red Panda of Nepal

**DOI:** 10.7717/peerj.3767

**Published:** 2017-09-06

**Authors:** Damber Bista, Saroj Shrestha, Ajaya Jang Kunwar, Sakshi Acharya, Shant Raj Jnawali, Krishna Prasad Acharya

**Affiliations:** 1 Red Panda Network, Baluwatar, Kathmandu, Nepal; 2 Kathmandu Center for Genomics and Research Laboratory, Gwarko, Lalitpur, Nepal; 3 ZSL Nepal, Baluwatar, Kathmandu, Nepal; 4 WWF Nepal, Baluwatar, Kathmandu, Nepal; 5 Department of Forests, Ministry of Forest and Soil Conservation, Government of Nepal, Babarmahal, Kathmandu, Nepal

**Keywords:** Red Panda, Endoparasites, Gastrointestinal parasites, Prevalence, Threat

## Abstract

Red pandas are known to be highly susceptible to endoparasites, which can have a prominent impact on the population dynamics of this endangered species. There are very limited published reports on prevalence and risk of parasites in wild populations of red panda, especially localized reports. This study attempts to provide an in-depth insight of the status of endoparasites in red pandas, which is critical for strengthening conservation efforts. A total of 272 fecal samples were collected through systematic sampling across the red panda distribution range in Nepal and coprological examination was completed using standard techniques. It was followed by an estimation of prevalence and mean intensity of parasites, as well as statistical analysis, which was carried out using R statistical software. Parasite prevalence was documented in 90.80% (*n* = 247) out of 272 samples examined which includes seven different species along with three genera of parasites belonging to Protozoans (3 species), Cestodes (1 genus, 1 species) and Nematodes (2 genera, 3 species). Nematodes predominated in all infected samples (87.62%). Prevalence of *Ancyclostoma duodenale* (*n* = 227, 70.06%), having a mean intensity of 3.45 ± 2.88 individuals per sample, was observed, followed by *Ascaris lumbricoides* (*n* = 19, 5.86%) and *Entamoeba histolytica* (*n* = 24, 7.41%). Eight variables for assessing the determinants of infestation were tested: protected areas; non-protected areas; aspect; elevation; slope; and distance to water sources, herding stations, and settlements. Only the settlement displayed significant association (*β* = −1534e−04, *t* =  − 2.192, *p* = 0.0293) though each parasite species displayed dissimilar association with different variables. This study indicates the urgent need of improving existing herding practice through habitat zonation, rotational grazing, medication of livestock, and prohibition of open defecation within and around red panda habitat.

## Introduction

The red panda (*Ailurus fulgens*) is the sole representative of the monotypic family *Ailuridae* (Roberts & Gittleman, 1984; Glaston, 1994) and a globally endangered species (Glatston et al., 2015). Disease can pose a serious threat to endangered species, occasionally causing sudden and unexpected local declines in abundance (Cleaveland et al., 2002). The mortality rate of the red panda is very high in the wild (∼65%), (Yonzon & Hunter, 1991) which can be attributed to several explanations, including predation by natural predators, dogs, killed by people, stress engendered by human disturbances provoking the mother to move their cubs frequently making them vulnerable to predation and physical injury, and parasitic infection, though the relative impact of any of these causes is yet to be assessed in the wild. Parasites can have a prominent impact on the population dynamics of wildlife and has emerged as a critical issue in conservation of threatened species (Thompson, Lymbery & Smith, 2010). Parasites can cause various problems for these species through the adverse effects of parasitism (Bliss, 2009). Parasites can affect host survival and reproduction directly through pathological effects (blood loss, tissue damage, spontaneous abortion, congenital malformations and death) and indirectly by reducing the host’s immunity and lessening the individual’s physical condition (Thawait, Maiti & Dixit, 2014).

Red pandas are known to be highly susceptible to gastrointestinal (GI) parasites (Grøndahl et al., 2005; Patterson-Kane et al., 2009; Lama et al., 2015), however very little information exists about GI parasite infection in this species, particularly in wild populations. While a number of parasitic diseases in captive pandas have been reported and described (Hu, 2001; Acharjyo, 2004; Qin et al., 2007; Bertelsen et al., 2010; Xie et al., 2011; Panayotova-Pencheva, 2013), there are relatively few published reports of parasitic diseases in wild populations. Bista & Paudel (2014) has speculated the death of one red panda in the Panchthar-Ilam-Taplejung corridor due to infection though the exact causes remain undetected.

Studies on gastrointestinal parasites in red panda are particularly scarce in Nepal. Limited localized studies have been done in Rasuwa district (Langtang National Park) (Sharma et al., 2016), Mugu district falling withing the Rara National Park (Shrestha & Maharjan, 2015) and Rolpa district (Lama et al., 2015). These studies do not wholly represent the scenario at the landscape and national levels and there is still a lack of baseline data for GI parasites infestation in wild red pandas. Therefore, this study attempts to provide in-depth insight of the GI parasitic status of red pandas of Nepal, which will be helpful in ensuring the survival of this threatened species through devising appropriate long-term conservation strategies.

## Materials and Methods

### Data/sample collection

Based on the previous available species presence data and environmental parameters including 19 bioclimatic variables (11 temperature and eight precipitation metrics) along with altitude, slope, aspect, and land cover, MaxEnt Modeling using MaxEnt version 3.3.3 k was completed to identify potential red panda habitat. Identified potential habitat was overlaid with grids to match the maximum red panda home range recorded in Langtang National Park (Fox, Yonzon & Podger, 1996). Grids which covered nearly 50% area of the identified habitat were considered for random selection up to 50% of the total grids. Each of the selected grids were further overlaid with six sub-grids (area = 1.6 km^2^), and again three grids were randomly selected as sampling sites. Through this method, a total number of 856 sub-grids were selected as sampling sites. However, only 557 sub-grids were covered due to limited time and financial constraints. Data was collected while traversing across a 1,364 km transect distance within 6,326 h by 35 field biologists, each of them accompanied with two assistants. Field work was carried out in June and July, 2016. GPS coordinates, elevation, and distance to the nearest water source, settlement, and herding station were also recorded in the field, whereas the aspect and slope were later retrieved using Q GIS.

The research approval was granted from the government authorities before the field work was carried out: Department of Forests #2072/73(1220) and Department of National Parks and Wildlife Conservation: 2072/073Eco237.

**Figure 1:**
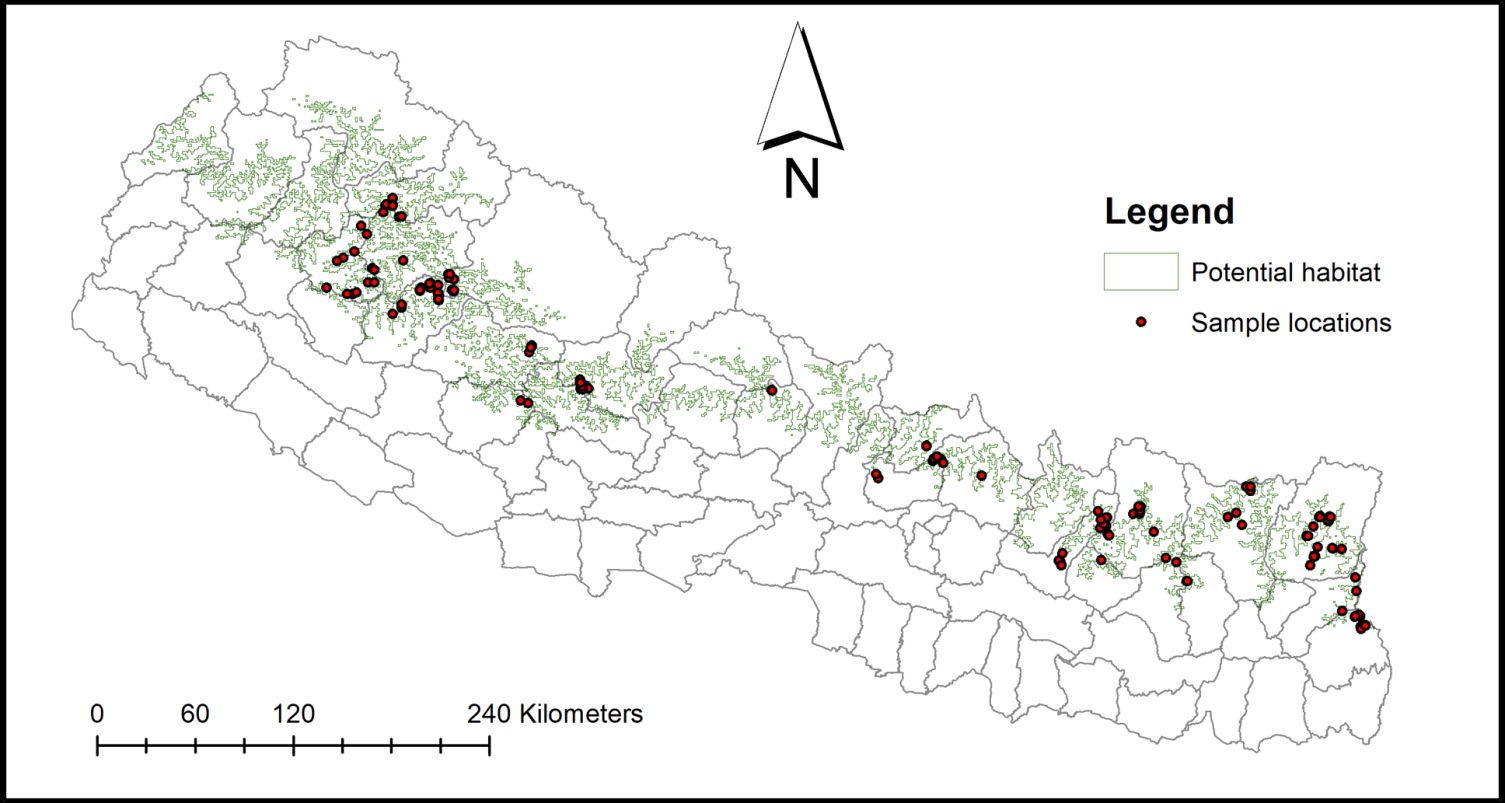
Sampling locations.

A total of 272 fecal samples (unspecified sex) from 21 districts (Fig. 1) were collected for GI parasite analyses. Fresh fecal samples with an intact outer mucosal layer were collected and preserved with 2.5% Potassium Dichromate in sterile, labeled 50 ml sampling tubes.

### Laboratory analysis

GI parasite analysis of fecal samples followed protocol developed and maintained by Centers for Disease Control and Prevention Division of Parasitic Diseases and Malaria by US Department of Health and Human Services (Mali et al., 2010). Initially, the flotation method was adopted for the examination of eggs and larvae of parasites. Each sample was mixed well and 5 ml of the fecal suspension was strained through wetted cotton gauze placed over a funnel into a 15 ml centrifuge tube. An additional 7 ml of 10% formalin was poured through the debris on the gauze. The tube was then centrifuged at 3,000 rpm for 5 min. Supernatant was decanted followed by an addition of 7 ml of 10% formalin to the pellet which was mixed thoroughly. The mixture was centrifuged at 3,000 rpm for 5 min. Followed that we poured the supernatant and added 7 ml of 10% formalin later by mixing, again mixture was centrifuged at 3,000 rpm for 5 min. We added 4 ml of ethyl acetate to the suspension and shook vigorously in an inverted position for 30 s. Again, the mixture was centrifuged at 3,000 rpm for 5 min, then freed plug of debris from top of the tube by ringing slides with an applicator stick. Later the top layer of supernatant was decanted and cotton tipped applicator was used to remove debris from sides of the centrifuge tube. Several drops of 10% formalin was added to re-suspend concentrated specimen.

A smear was prepared with 1–2 drops of specimen per slide and warmed at 60 °C until dried and then fixed with absolute methanol for 30 s. The slide was stained with Kinyoun’s carbol fuchsine for one minute. The slide was rinsed briefly with distilled water and drained. Next it was de-stained with acid alcohol for 2 min, afterward rinsed briefly with distilled water and drained. Similarly, it was counter stained with Malachite green for 2 min, rinsed briefly with distilled water and drained. Once again, the slide was warmed at 60 °C for about 5 min until dried and mounted with a cover slip using desired mounting media. The slide was examined 200–300 fields using 40× or higher objectives.

We used photos of different forms: egg and larva, ova, cyst and adult of possible parasites of existing literatures to compare the parasites of red panda scats. The size of the eggs, oocyst, and cyst were measured using an ocular micrometer. Identification of parasitic oocyst, cyst, egg and larva were done on the basis of shape and size along with published literature (Barutzki & Schaper, 2009; Bhir, 1998; Canadian Parasitology Expert Panel, 2009; Brianti et al., 2013). The severity level of parasites was categorized into three groups based on their number per cover slip, viz. (+) for 1–3, (+ +) for 4–10 and (+ +  +) for more than 10.

Descriptive analysis was done by estimating the prevalence and mean intensity of parasites. Prevalence was examined by dividing the number of specific samples infected by the total number of infected samples. Whereas, the mean intensity was estimated by dividing the total number of parasites of a particular species by the total number of samples infected with that particular species (Bush et al., 1997). Statistical analysis was carried out using R statistical software (R Core Team, 2016). Forward selection of the model was done using step AIC function in MASS package (Venables & Ripley, 2002) of R. Poisson distribution regression model selection was done using AIC value (Burnham & Anderson, 2004). We had considered eight different variables as distribution determinants of parasites, viz. protected areas; non-protected areas; aspect; elevation; slope; and distance to water sources, herding stations, and settlements. Some models were found to violate the assumption of Poisson distribution model (i.e., mean = variance) thus for those models quasi Poisson regression modeling was used to incorporate the impact of over dispersion on estimation of standard error.

## Results

Parasite prevalence was identified in 90.80% (*n* = 247) out of 272 samples examined while uninfected samples were recorded as 9.20% (*n* = 25) samples. Seven different species along with three additional genera of parasites were identified, which belonged to Protozoans (3 species), Cestodes (1 genus, 1 species) and Nematodes (2 genera, 3 species). Nematodes predominated in all the infected samples (87.62%) followed by protozoans (8.69%) and cestodes (3.67%). The majority of samples were observed to be infected with hookworm i.e., *Ancyclostoma duodenale* (*n* = 227, 70.06%), having a mean intensity of 3.45 ± 2.88 individuals per sample followed by *Entamoeba histolytica* (*n* = 24, 7.41%) and *Ascaris lumbricoides* (*n* = 19, 5.86%). In addition, the *Strongyloides stercorallis* (*n* = 13, 4.01%) and *Taenia* spp. (*n* = 7, 2.16%) were also prevalent in the infected. The other two genera (*Strongyloides* spp*., n* = 2* and Trichostrongylus* spp., *n* = 1) and three species including *Diphyllobothrium latium* (*n* = 4), *Sarcocystis calchasi* (*n* = 1) and *Cyclospora cayetanensis* (*n* = 1) were documented in four or less number of samples (Table 1).

**Table 1:** Prevalence of gastrointestinal parasites in red panda.

Parasites name	Number of samples	Prevalence (%)	Mean	SD
*Ancylostoma duodenale*	227	70.06	3.45	2.88
*Ascaris Lumbricoides*	19	5.86	5.21	1.56
*Strongyloides stercoralis*	13	4.01	4.00	0.24
*Trichostrongylus* spp.	1	0.31	2.00	0.12
*Strongyloidesspp.*	2	0.62	2.00	0.09
*Cyclospora cayetanensis*	1	0.31	8.00	0.24
*Entamoeba Histolytica*	24	7.41	3.33	0.89
*Sarcocystis calchasi*	1	0.31	6.00	0.21
*Taenia* spp.	7	2.16	6.43	1.21
*Diphyllobothrium latum*	4	1.23	4.75	0.51

Out of the total infected samples (*n* = 247), the majority (80.97%) were recorded to be infected with a single parasite, while multiple infections with two or more than two parasites were observed in 19.03% (*n* = 47) of the samples. These parasites were recorded in five different forms in different order, viz. larval form (57.84%) followed by ova (25.93%), cyst (8.16%), adult worm (4.17%) and egg (3.90%). The *Ancyclostoma duodenale* was found in all five different forms while other parasites were recorded to be either in one or up to three different forms.

Parasitic infestation was recorded throughout the entire red panda range area of Nepal. Out of the eight variables considered for assessing the determinants of infestation, only the distance to settlement displayed significant association when fitted with the quasi-poison regression model (*β* =  − 15.4e − 04, *t* =  − 2.192, *p* = 0.0293) which indicated that the prevalence of endoparasites increases nearby settlements. However, each of these parasite species displayed dissimilar association with different variables. Detection of five parasites was very low (number of infected samples ≤ 4), which did not allow to draw any inference. Therefore, only the five parasites with remarkable prevalence (number of infected samples ≥ 7) were analyzed to see their association with different determinants.

The best fit model was found to be slightly over dispersed for *Ancyclostoma duodenale*, *Entamoeba histolytica*, *Ascaris lumbricoides*, and *Strongyloides stercorallis,* therefore, quasi Poisson modeling had to be applied in order to compensate the over dispersion effect in the model. The detection of *Entamoeba histolytica* was found to be influenced by aspect, slope, the distance from the herding stations, and interaction of aspect with slope. The quasi Poisson distribution regression model also indicated a negative relationship of *Ascaris lumbricoides* with the slope and distance from settlement areas. The model further indicated a positive association of prevalence of *Strongyloides stercorallis* with elevation. The best fit quasi model for tape worm was observed to be highly under-dispersed so it was updated with the addition of some other variables resulting insignificant association with all the variables. Likewise, the model failed to show a significant association of *Ancyclostoma duodenale’s* prevalence with any of the variables (*β* =  − 9.94E − 01, *t* =  − 1.59 and *p* = 0.11) indicating these parasites as generalist species (Table 2).

**Table 2:** Variables affecting the prevalence of GI parasites.

Variables	Estimate (*β*)	Std. error	*t* value	*Pr*(>|*t*|)
*Ancyclostoma duodenale*				
(Intercept)	−9.94E−01	6.25E−01	−1.59	0.11302
*Entamoeba histolytica*				
Intercept	−3.754857	0.97374	−3.86	0.00012
Herding station	−0.000597	0.000168	−3.55	0.00039
Aspect	0.013955	0.00421	3.315	0.00092
Slope	0.073975	0.027849	2.656	0.0079
Aspect:slope	−0.000319	0.000133	2.391	0.01679
*Ascaris lumbricoides*				
(Intercept)	1.386668	0.514031	2.698	0.00751
Settlement	−0.001223	0.000414	−2.96	0.00345
Slope	−0.04097	0.020788	−1.97	0.04995
*Strongyloides stercorallis*				
(Intercept)	−13.05	4.28	−3.05	0.00257
Elevation	0.002692	0.00135	1.995	0.04729
*Taenia* spp.				
Intercept	0.1310452	0.7191754	(*z* value) 0.182	0.8554100

## Discussion

With the representation of 272 samples collected through systematic sampling from the entire red panda range of Nepal, these results are highly representative of the Nepal population. Prevalence of parasites in 90.80% of the examined samples with a very high load of parasites representing three genera and seven species falling under three groups, viz. protozoa, nematodes and cestodes indicates very high transmission within the red panda population. Previous studies on the red panda in Langtang National Park, Rara National Park and Rolpa district in Nepal have also documented the presence of trematode in addition to those three groups, but presence of trematode remained undetected in this study (Sharma et al., 2016; Shrestha & Maharjan, 2015; Lama et al., 2015). Prevalence of nematodes remained the highest (87.58%) which could be attributed to the direct life cycle of nematodes without involvement of any intermediate host, which is in accordance with the findings of Thawait, Maiti & Dixit (2014). The source of transmission of these parasites could also be due to the habitat encroachment by co-grazing livestock. Morgan et al. (2006) has also reported that gastrointestinal nematode infections were the main risks associated with infections from livestock.

Altogether, 18 parasites recorded in former studies remained undetected (Table 3) in the present study (Sharma et al., 2016; Shrestha & Maharjan, 2015; Lama et al., 2015; Wang et al., 2015; Pradhan et al., 2011; Bertelsen et al., 2010) with the record of three new parasites viz., *Thichostrongylus* spp., *Strongyloides* and *Sarcocystis calcasi*. Prevalence of different genera and species vary with the time of year (Vimalraj, Partridge & Selvamurugan, 2014), which may account for the lack of uniformity in the species accounted in various studies as the survey time was slightly different between each study.

**Table 3:** GI parasites undetected in present study.

S.N.	GI parasites	Studies detecting presence
1	*Cryptosporidium sp.*	^***^,^****^
2	*Baylisascaris schroederi*	^**^,^***^
3	*Crenosoma* sp.	^******^
4	*Metastrongylus* sp.	^**^, ^******^
5	*Oxyuris* sp.	^**^
6	*Moniezia* sp.	^*^,^**^
7	*Trichuris* sp.	^*^,^**^,^***^
8	*Eimeria* sp.	^**^
9	*Toxocara* sp.	^**^,^*****^
10	*Toxascaris* sp.	^**^,^*****^
11	*Fasciola sp.*	^*^,^*****^
12	*Dictyocaulus sp.*	^*****^
13	*Schistosoma sp.*	^*****^
14	*Trichomonas sp.*	^*****^
15	*Crenisomatidae sp.*	^**^
16	*Angiostrongylus sp.*	^**^,^***^,^*****^,^******^
17	*Capillaria sp.*	^*^
18	*Spirurida sp.*	^*^

**Notes.**

* Sharma et al. (2016).

** Shrestha & Maharjan (2015).

*** Lama et al. (2015).

**** Wang et al. (2015).

***** Pradhan et al. (2011).

****** Bertelsen et al. (2010).

The regression model showed a negative association of parasite prevalence with the distance to settlement indicating an anthropogenic source of infestation from associated livestock and pet animals. Prevalence of these parasites have been recorded in yak (Wiener, Jianlin & Ruijun, 2003); cattle (Vimalraj, Partridge & Selvamurugan, 2014); and domesticated cat (Murphy & Spickler, 2013), which supports this finding. Sharma et al. (2016) has also concluded similar findings indicating livestock as a competent agent of transmission of parasites to red pandas. Furthermore, Lama et al. (2015) had also specified pet animals like dogs and cats as potential agents of transmission.

Present study manifested diverse nature of distribution for *Ancyclostoma duodenale* with the highest prevalence amongst all identified parasites (*n* = 227, 70.06%). This parasite was earlier reported in red panda from Nepal (Sharma et al., 2016; Shrestha & Maharjan, 2015). It can be transmitted through fecal-oral, cutaneous penetration, and causes high mortality in animals (Hotez et al., 2004). Infections have also been recorded in different species of wild carnivores like wild dog, wolf, jackal, fox, jungle cat, clouded leopard, tiger, civet cat, honey badger, fishing cat, leopard, and mongoose (Pythal et al., 1993; Nashiruddullah & Chakraborty, 2001; Chowdhury, 2001). Animals most commonly remain asymptomatic, but diarrhea and anemia can be caused by infection, which can be fatal to cubs. Necropsy of a tiger carcass in Karnatak state, India had also revealed petechial hemorrhages due to infection of *Ancyclostoma* spp. from duodenal region (Ananda, Ganganaik & Kavitha Rani, 2014). Necropsy of another female tiger carcass also revealed *Ancyclostoma* spp. fully occupying the intestine. Sources of transmission of this parasite could be any of livestock or other sympatric species (Ananda, Ganganaik & Kavitha Rani, 2014).

*Ascaris lumbricoides* remained another dominant nematode (*n* = 19, 5.86%) in this study, which was also recorded in red panda feces in previous studies (Sharma et al., 2016; Shrestha & Maharjan, 2015; Pradhan et al., 2011). Achhami, Sharma & Bam (2016) also reported high prevalence of *Ascaris* in musk deer, a sympatric species from Langtang National Park. In another two studies, these parasites were also reported in Chauri (hybrid of cow and yak) from LNP (Sharma et al., 2016) and Ramechap district (Shrestha & Bindari, 2013) indicating the source of transmission towards livestock or vice versa. However, higher prevalence of *Ascaris lumbricoides* to nearby the settlement (*β* =  − 0.001223, *t* =  − 2.96 & *p* = 0.003), and low slope areas (*β* =  − 0.04097, *t* =  − 1.97 & *p* = 0.04995) indicate towards the domestic animals as infection source. Heavy infestation of *Ascaris lumbricoides* may cause severe complications due to bloody diarrhea in herbivores (Mass, 2007).

Other three nematodes documented in this study were the *Strongyloides stercorallis* (*n* = 13, 4.01%); *Strongyloides* spp. (*n* = 2, 0.62%) and *Trichostrongylus* spp. (*n* = 1, 0.31%). The occurrence of *Strongyloides* spp. and *Trichostrongylus* spp. seem to be accidental cases from contamination through human, cat, and dog feces. Out of those, *Strongyloides stercorallis* interestingly showed preference (*β* = 0.002692, *t* = 1.995 & *p* = 0.047) for higher elevation (3,595 ± 252.91 m). Traditional transhumance grazing method is practiced throughout much of the high-altitude in Nepal. Annual cycle of transhumance migration of grazing animals begins from mid-March, moving from sub-tropical grazing areas (2,000–3,500 m) to temperate pasture by mid-May, and then remains at the higher altitudes until September (Oli, 2008).This might be a possible reason for the prevalence of *Strongyloides stercorallis* was found in samples from higher elevations that mostly remained occupied with the livestock during the sampling period. *Strongyloides* spp. has also been reported in red pandas from Rara National Park and Langtang National Park (Sharma et al., 2016; Shrestha & Maharjan, 2015). These parasites can cause diarrhea, anorexia, weight loss, and dyspnea, which has been already reported in deer (Ezenwa, 2003; Meshram et al., 2008). This nematode is pathologically very important as it can result ocular, neural, and Visceral Larvae Migrans (VLM) causing blindness, loss of muscle control, hepatomegaly and coma (Shrestha & Maharjan, 2015) when infecting a non-natural host (Sharma et al., 2016). The VLM could be one of the causative factors of high mortality rate in red panda as a similar finding has been documented in China where 50 ± 10.2% (12/24) panda deaths were recorded because of VLM from 2001 to 2005 (Zhang et al., 2008). Further study is necessary to quantify red panda susceptible to this particular disease.

Three different genera of protozoans reported during the coprological examination are also very common in livestock which overlaps red panda habitat in the region. *Entamoeba histolytica* documented as the second most prevalent parasite (*n* = 24, 7.41%), however is lower than the previous report (62.79%) from RNP, western Nepal (Shrestha & Maharjan, 2015). These parasites have shown significant association with the distance to herding station (*β* =  − 0.000597, *t* =  − 3.55 & *p* = 0.0003) indicating livestock as a major source of transmission. *Entamoeba histolytica* which can cause amoebic dysentery, were previously reported in red panda faces in the RNP (Shrestha & Maharjan, 2015). The source of these parasites could also be attributed to human feces contaminating water sources because of open defecation as these are one of the most common GI parasites in Nepal (Shakya et al., 2009).

Infestation of *Cyclospora cayetanensis* was also observed to be remarkable (*n* = 1, 0.31%) which was previously reported in red panda feces (Lama et al., 2015). These parasites have already been reported in humans, dogs, and primates in Nepal (Chu et al., 2004; Li et al., 2017). These records indicate accidental contamination from the feces of human, dogs, and primates to the red panda; however the red panda can also serve as a natural reservoir host, which needs further investigation. These parasites may pose detrimental effects to red panda survival because of diarrhea, and weight loss which are also common during the infection of humans (Soave, 1996).

This study reported *Sarcocystis* spp. for the first time in red pandas, albeit only one sample was infected. These parasites have a 2-host life cycle, with carnivores e.g., dog, wolf, fox, cat, as definitive hosts and herbivores e.g., horse, sheep, monkey as intermediate hosts (Dubey, 1976), which can be the causative agent of highly fatal protozoal encephalitis (Nanako et al., 2015).

One genera (*Taenia* spp., *n* = 7) and one species of cestodes (*Diphyllobotium latum*, *n* = 1) were also recorded in this study though their presence was detected in small fraction. The *Taenia* spp. was earlier reported in red panda feces (Sharma et al., 2016) but the latter was a new recording for red panda. A cestode was also detected in red panda feces in Rolpa district, Nepal though it remained unidentified (Lama et al., 2015). Infection from cestode has been documented in captive populations of lion, leopard, hyaena, and ratel in Nandan Kannan Zoo in India (Thawait, Maiti & Dixit, 2014) and this parasite species has remained very common in tiger, leopard, and sloth bear in Karnataka state, India resulting in enteritis in sloth bear (Muraleedharan, 2015). Infection with *Taenia* spp. could be fatal to red pandas as necropsy of a dead tigress revealed *Taenia* spp. and *Ancyclostoma* spp. clogging its intestine. Interestingly, the *Taenia* spp. did not show association with any distribution determinants (*β* = 0.1310452, *z* = 0.182 & *p* = 0.8554100). *Diphyllobothrium* spp. was found in dog stool samples, and in the stool of ethnic communities depending on agriculture and fishing in Nepal (Devleesschauwer, 2015). This evidence again is indicative of accidental contamination through human or dog feces.

Animal behavior is the critical factor to be considered while identifying the potential factors influencing parasite transmission (Stephanie, 2013). Based on red panda’s behavior, there are some potential modes of transmission. Out of them, consumption of contaminated food and water is the most likely mode as infective stages of parasites e.g., eggs, cysts, oocysts and larval forms passed in feces of livestock and other sympatric wild animals that may be there. However, the chances of contamination of food are comparatively lower as the red panda mostly forage from the trees which are out of reach to many sympatric ungulate, canid, and livestock. Nevertheless, the foraging leaves could be contaminated through the droppings of infected arboreal primates, felids, and birds. Their feeding behavior is not same all the year. They also come down on ground to feed on bamboo shoots especially during the rainy season which is more likely to be contaminated with the parasites. Association of parasite prevalence with the proximity to settlement also indicated the possible contamination of food and water sources their human feces as most of the parasites viz., *Ancyclostoma duodenale*; *Entamoeba histolytica; Ascaris lumbricoides; Sarcocystis calchasi*; Diphyllobothrium latium and *Cyclospora cayetanensis* observed in this study are also common in human.

Direct contact with other individuals is another possible mode of transmission. Although the red panda is a solitary mammal, a male comes in contact with several females during mating season which may be a possible occurrence of transmission through direct contact. The spreading of parasites through infected milk from a mother to her cubs is also possible. Contaminated droppings of a red panda could be the source of infection for other individuals as mostly they defecate on trees where the droppings could remain preserved for several months unless it is washed off by the precipitation. The very interesting behavior of the red panda is licking or nuzzling the urine marks and feces of conspecifics to track their territories which may also be another common mode of parasite transmission (Zhang et al., 2007). Lama et al. (2015) had also reported this transmission mode in red pandas.

Direct contact with the contaminated sources e.g., animal droppings, dead animals, and the tree/branch surfaces may also contribute to infection as some larval stage of parasites can directly penetrated through the skin. Ectoparasites like fleas, ticks and mites may also facilitate the transmission through establishing direct contact of parasites with animals.

## Conclusion

This study provides an overview of GI parasites infestation in red pandas at the national level and could be used as baseline to assess the status of endoparasite for future works. In general, the parasitic strains in red pandas across their range in Nepal showed very detrimental symptoms. The result of this work has also raised a few questions regarding the prevalence, risk, and implication of endoparasites in red pandas in Nepal. Analysis based on laboratory work limited identification up to generic level for some parasites. A genetic approach should be taken for identifying up to species level influence. This study is also limited to the fecal sample analysis of red pandas. Fecal samples of livestock could not accompany this work which could have revealed better information on host-parasite relationship and mode of transmission. Therefore, incorporation of coprology of co-grazing livestock, and red panda during different seasons is recommended for further study which will provide better insight on the GI status in red pandas as well as livestock and other sympatric species. Furthermore, a thorough ecological study on host-parasite relationship is also critical for better understanding the risk and conservation implication for red pandas. A parasite concern of this degree could critically undermine the heath of the animals. As our findings suggest, livestock herding is a detrimental threat to red panda conservation as they seem to be frequent carriers of GI parasites, and we should manage herding practices in such a manner that the impact could be minimized. Habitat zonation, rotational grazing, proper medication of livestock and dogs, use of toilets in herding sheds and the settlement vicinity to the red panda habitat could be helpful in preventing transmission of zoonotic parasites.

##  Supplemental Information

10.7717/peerj.3767/supp-1 Table S1 GI Parasites detected in fecal samples

10.7717/peerj.3767/supp-2 Figure S1 Cysts of *Cryptosporidium parvum*

10.7717/peerj.3767/supp-3 Figure S2 Ova of *Diphyllobothrium latum*

10.7717/peerj.3767/supp-4 Figure S3 Ova of *Ascaris lumbricoides*

10.7717/peerj.3767/supp-5 Figure S4 Ova of *Ancyclostoma duodenale*

10.7717/peerj.3767/supp-6 Figure S5 Strongyloides stercoralis

10.7717/peerj.3767/supp-7 Figure S6 *Taenia* sp

10.7717/peerj.3767/supp-8 Figure S7 Trophozoite of *Entamoeba histolytica*

## Additional Information and Declarations

### Field Study Permissions

The following information was supplied relating to field study approvals (i.e., approving body and any reference numbers): The research approval was granted from the government authorities before the field work was carried out: Department of Forests #2072/73 (1220) and Department of National Parks and Wildlife Conservation: 2072/073Eco237.

### Data Availability

The following information was supplied regarding data availability: The raw data has been uploaded as Supplemental Files.
