# Supplementary figures and images for: Status of gastrointestinal parasites in Red Panda of Nepal
Source: PeerJ. 2017 Sep 6;5:e3767. doi: 10.7717/peerj.3767 (PMC5591639; doi:10.7717/peerj.3767)

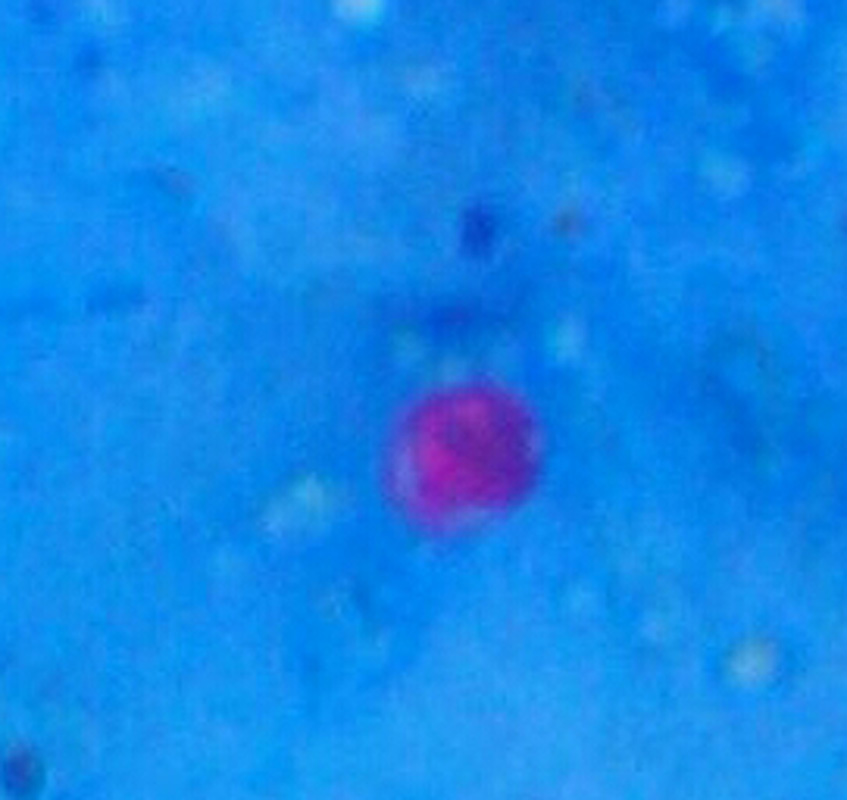

Supplement: Figure S1 [file peerj-05-3767-s002.jpg]

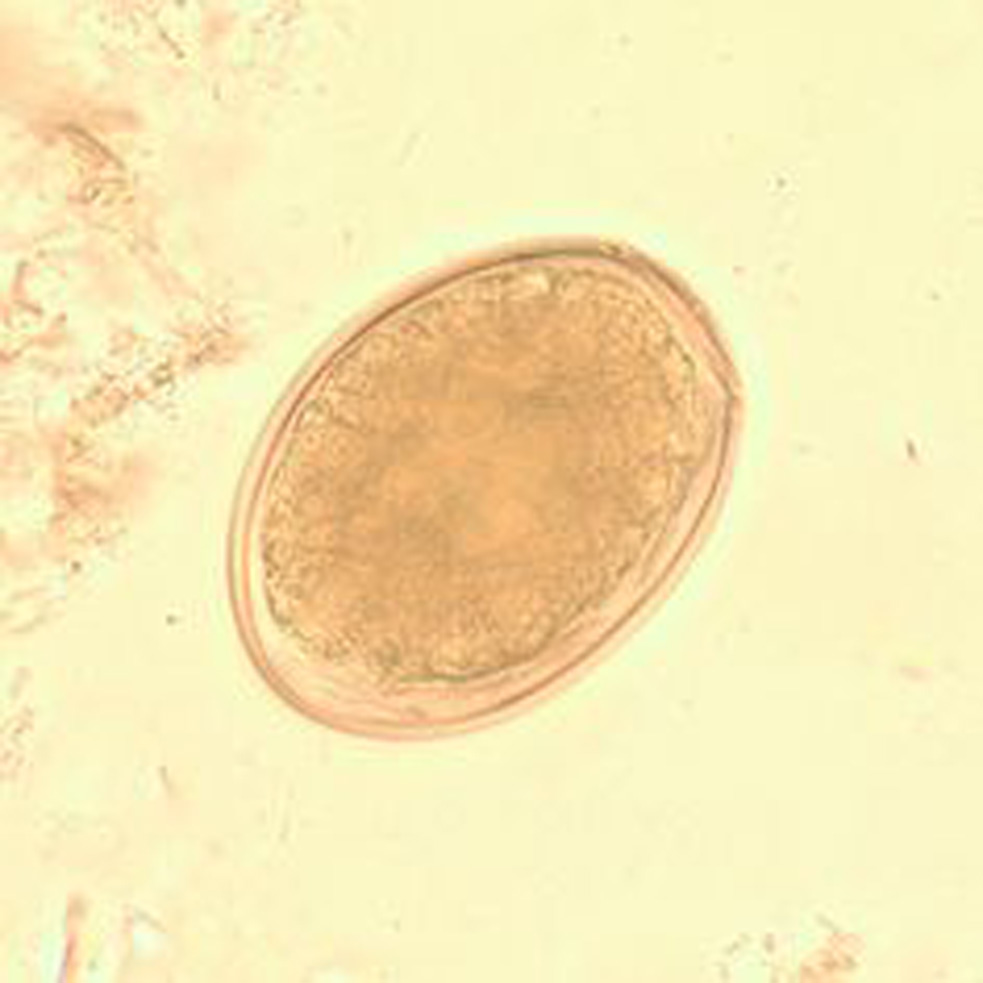

Supplement: Figure S2 [file peerj-05-3767-s003.jpg]

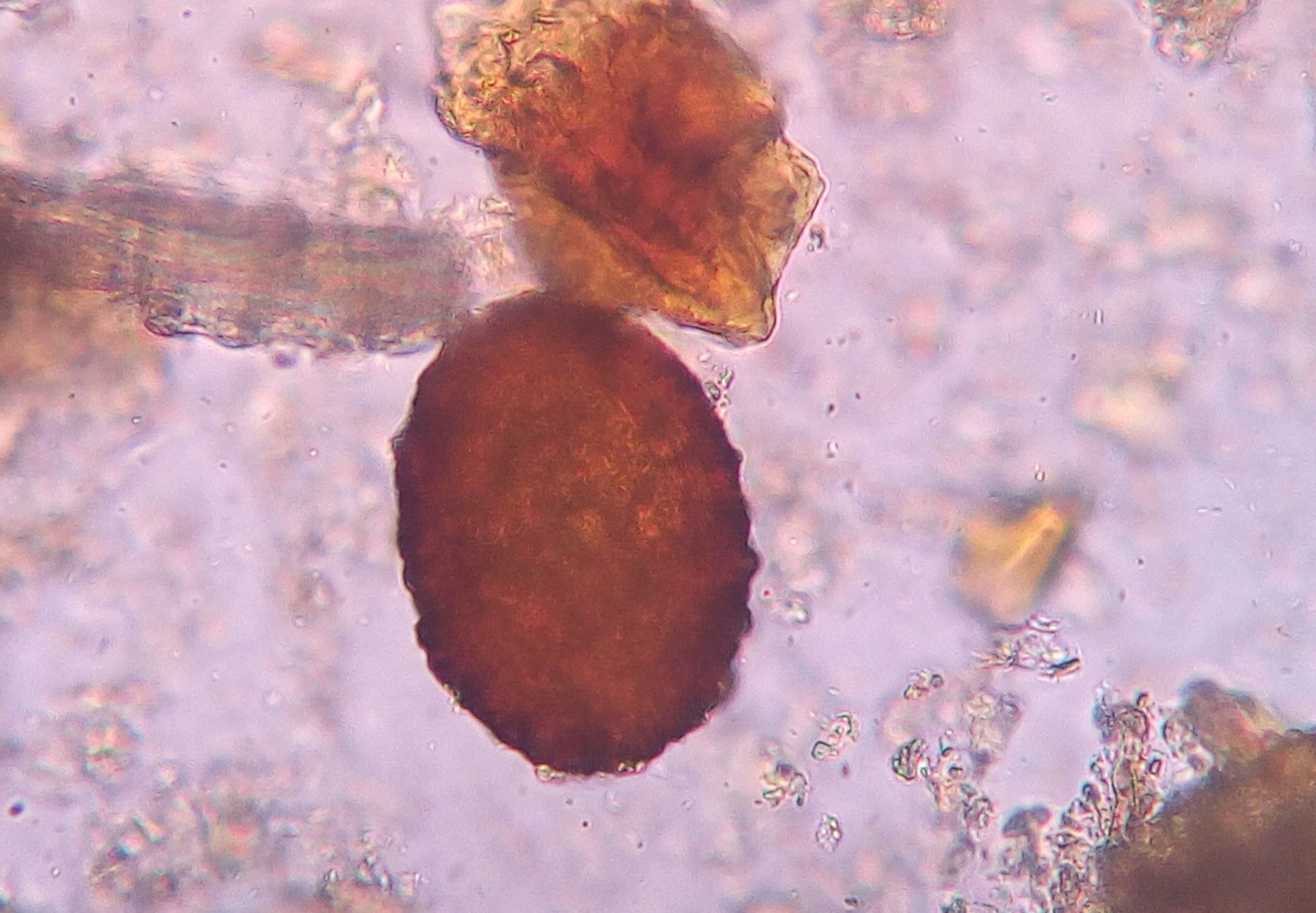

Supplement: Figure S3 [file peerj-05-3767-s004.jpg]

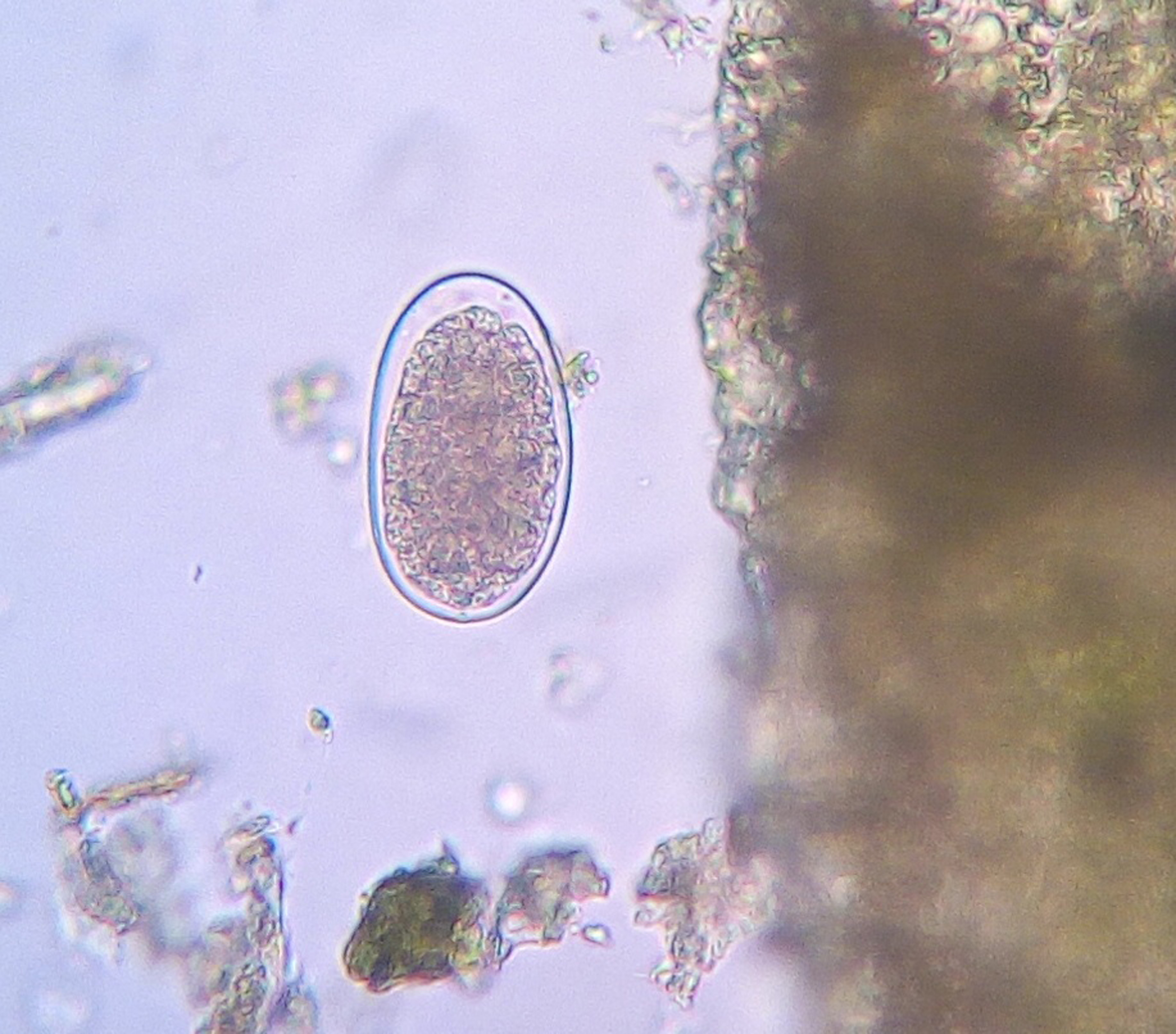

Supplement: Figure S4 [file peerj-05-3767-s005.jpg]

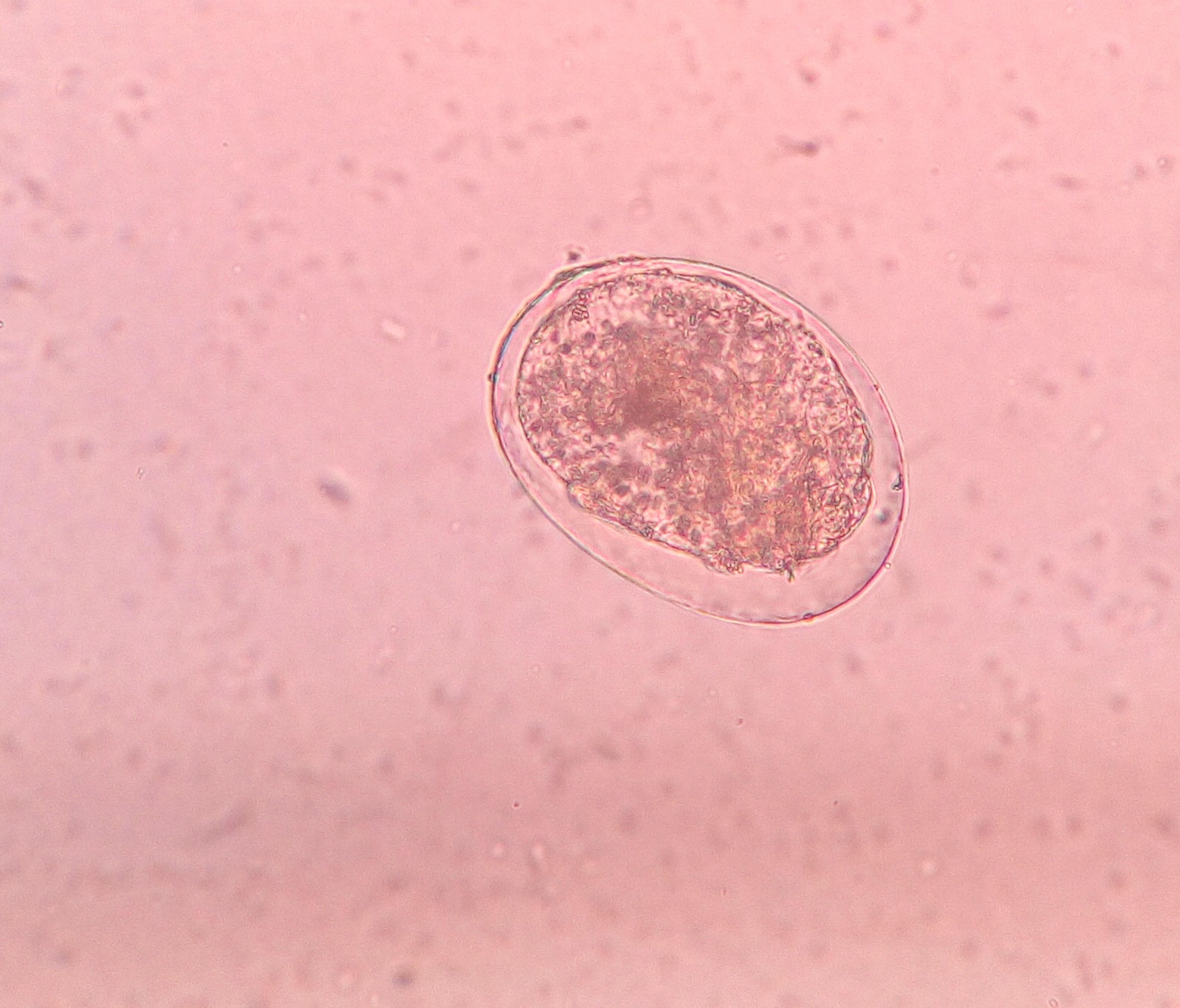

Supplement: Figure S5 [file peerj-05-3767-s006.jpg]

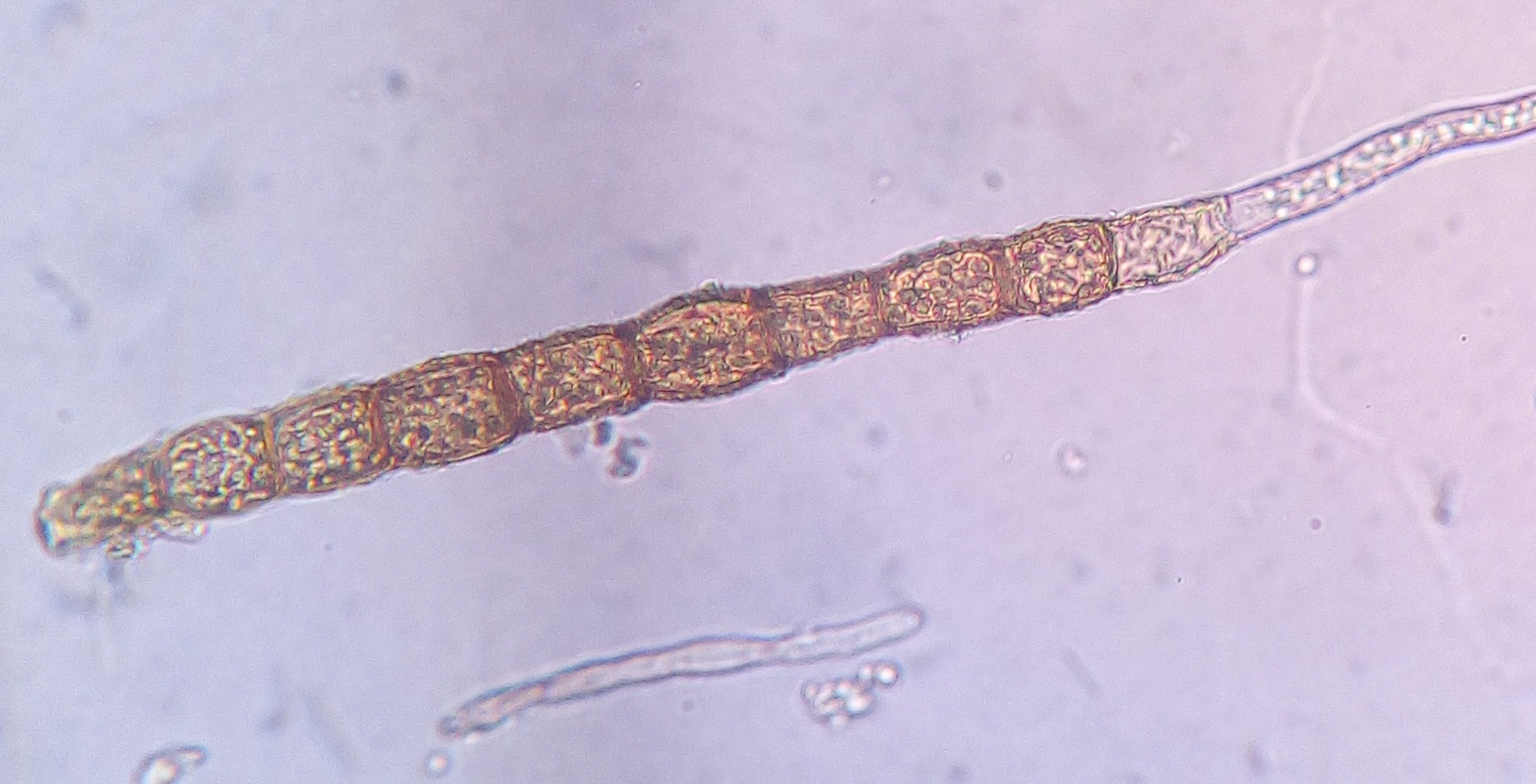

Supplement: Figure S6 [file peerj-05-3767-s007.jpg]

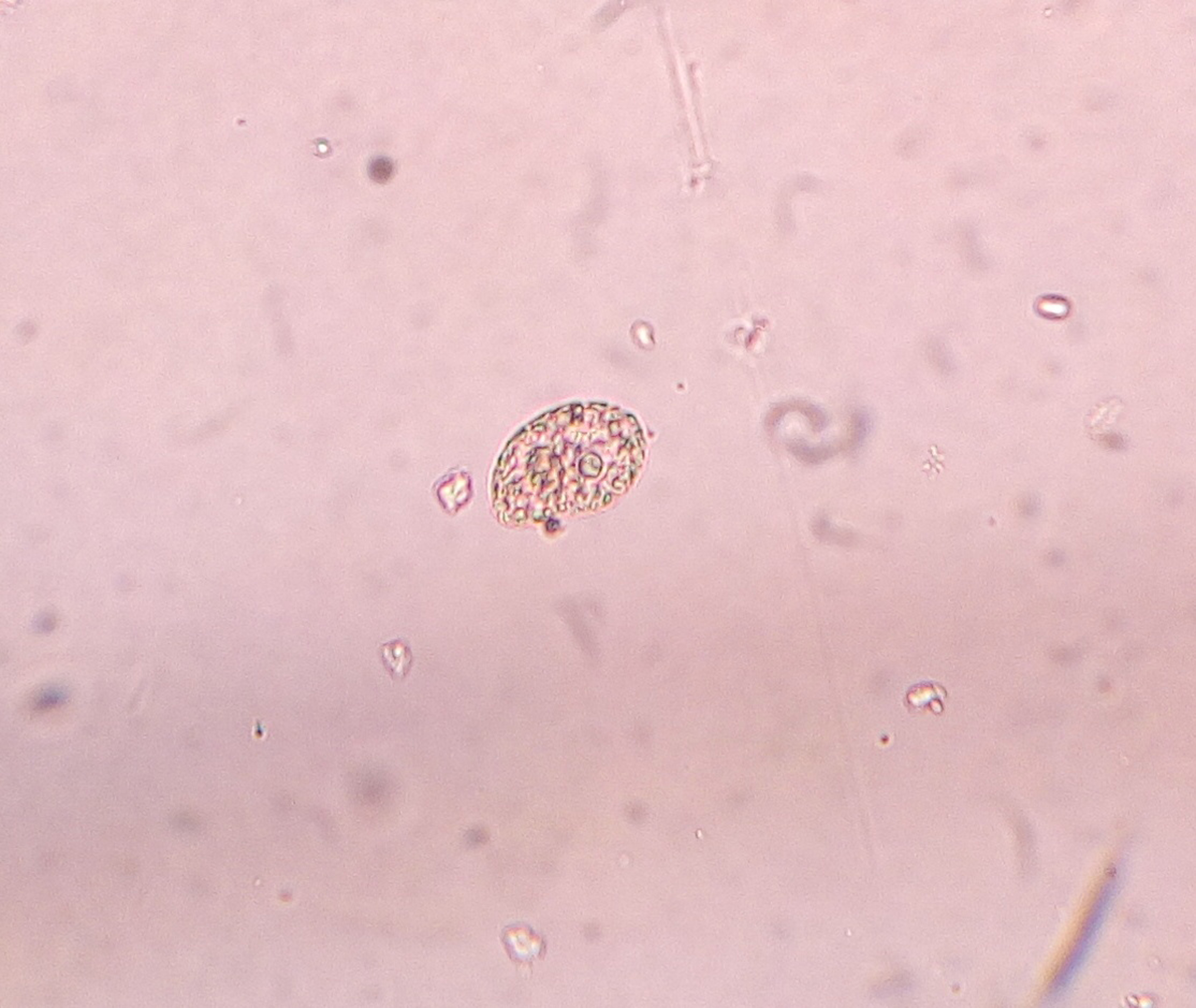

Supplement: Figure S7 [file peerj-05-3767-s008.jpg]
